# Sounds Move a Static Visual Object

**DOI:** 10.1371/journal.pone.0012255

**Published:** 2010-08-19

**Authors:** Wataru Teramoto, Souta Hidaka, Yoichi Sugita

**Affiliations:** 1 Department of Psychology, Graduate School of Arts and Letters, Tohoku University, Sendai, Miyagi, Japan; 2 Research Institute of Electrical Communication, Tohoku University, Sendai, Miyagi, Japan; 3 Department of Psychology, Rikkyo University, Niiza, Saitama, Japan; 4 Neuroscience Research Institute, National Institute of Advanced Industrial Science and Technology (AIST), Tsukuba, Ibaraki, Japan; University of Sydney, Australia

## Abstract

**Background:**

Vision provides the most salient information with regard to stimulus motion, but audition can also provide important cues that affect visual motion perception. Here, we show that sounds containing no motion or positional cues can induce illusory visual motion perception for static visual objects.

**Methodology/Principal Findings:**

Two circles placed side by side were presented in alternation producing apparent motion perception and each onset was accompanied by a tone burst of a specific and unique frequency. After exposure to this visual apparent motion with tones for a few minutes, the tones became drivers for illusory motion perception. When the flash onset was synchronized to tones of alternating frequencies, a circle blinking at a fixed location was perceived as lateral motion in the same direction as the previously exposed apparent motion. Furthermore, the effect lasted at least for a few days. The effect was well observed at the retinal position that was previously exposed to apparent motion with tone bursts.

**Conclusions/Significance:**

The present results indicate that strong association between sound sequence and visual motion is easily formed within a short period and that, after forming the association, sounds are able to trigger visual motion perception for a static visual object.

## Introduction

Both vision and audition provide important information about a moving object. It has been shown that, although vision provides the most salient information with regard to stimulus motion, audition provides important cues that affect visual motion perception [1]–[6]. For example, a blinking visual stimulus appears as lateral motion when the flash onset is synchronized to an alternating left-right sound source [5], [6]. Correspondingly human fMRI studies suggested a modulatory, but not driving, effect of auditory motion on MT+ responses [7], [8].

We found that strong association between sound sequence and visual motion is easily formed within a short period and that, after forming the association, sounds are able to trigger visual motion perception for a static visual object. Two white circles placed side by side were presented in alternation. The onsets of the two circles were synchronized to a tone burst of high and low frequency, respectively. After exposure to the visual apparent motion with tone bursts for 3 min, a circle blinking at a fixed location was perceived as lateral motion in the same direction as the previously exposed apparent motion, when the flash onset was synchronized to the tones (Video S1 and S2 for demonstration). Furthermore, the effect lasted for a considerably long time, at least for a few days. We quantified the strength of the illusory motion by a motion nulling procedure with the method of constant stimuli.

## Results

**Figure 1:**
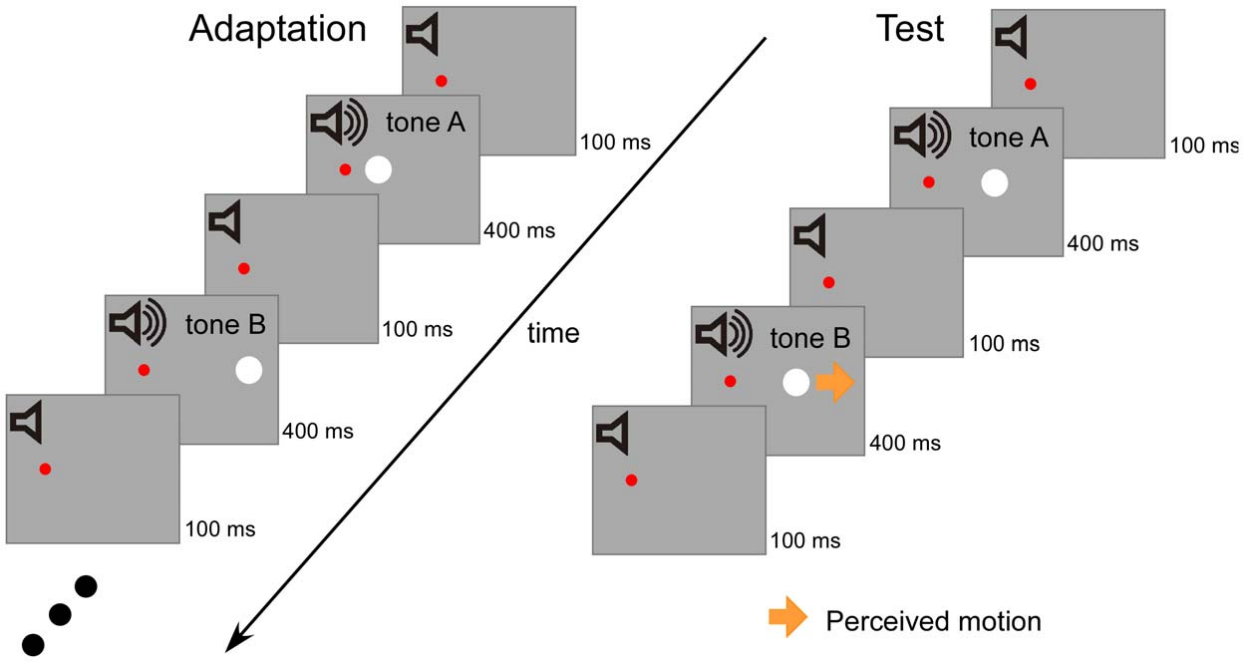
Experimental design. In an adaptation phase, observers were exposed to visual apparent motion for 3 min in which two white circles placed side by side were presented in alternation. The onset of leftward circle was accompanied by a tone A and the rightward circle a tone B. In a test phase, a white circle was presented twice. The circle was perceived as lateral motion in the same direction as the previously exposed apparent motion, when the onset of the circle was synchronized to tones of alternating frequencies. When the onset of the first circle was synchronized to the tone A and the second circle the tone B, the circle appeared to move from left to right. The strength of this illusory motion was quantified by a motion nulling procedure.

The strength of the illusory visual motion was quantified before and after the 3 min exposure to apparent motion with tone bursts (Fig. 1). In the exposure phase, two white circles (5.12 cd/m^2^, 1.0° in diameter) were presented in alternation at 7.5° and 12.5° to the right of the red fixation (17.47 cd/m^2^, 0.4° in diameter), respectively. The duration of each circle was 400 ms and stimulus onset asynchrony (SOA) was 500 ms. The onset of the rightward circle was synchronized to a tone burst of high (2 kHz) frequency and the leftward circle low (500 Hz) frequency for three observers. The opposite pairing was employed for the remaining three observers. Tone bursts (50 ms in duration with 5 ms rise and fall time) were presented through headphones. Observers were asked to keep looking at the fixation. In test sessions, a white circle (400 ms in duration) was presented twice with 500 ms of SOA, synchronized with two tone bursts (50 ms duration). The visual stimulus was displaced 0.12°, 0.24°, 0.48°, or 0.96° from left to right or vice versa. The center of these two positions was at 10.0° right from the fixation. The frequency of tones were alternated from 500 Hz to 2 kHz or 2 kHz to 500 Hz. The no-sound condition was also included. The observers were asked to judge whether the visual stimulus moved leftward or rightward. To determine the amount of visual displacement that corresponded to a point of subjective stationarity (PSS), we estimated the 50% point (the point of subjective equality) by fitting a cumulative normal-distribution function to each individual's data using a maximum likelihood curve fitting technique.

**Figure 2:**
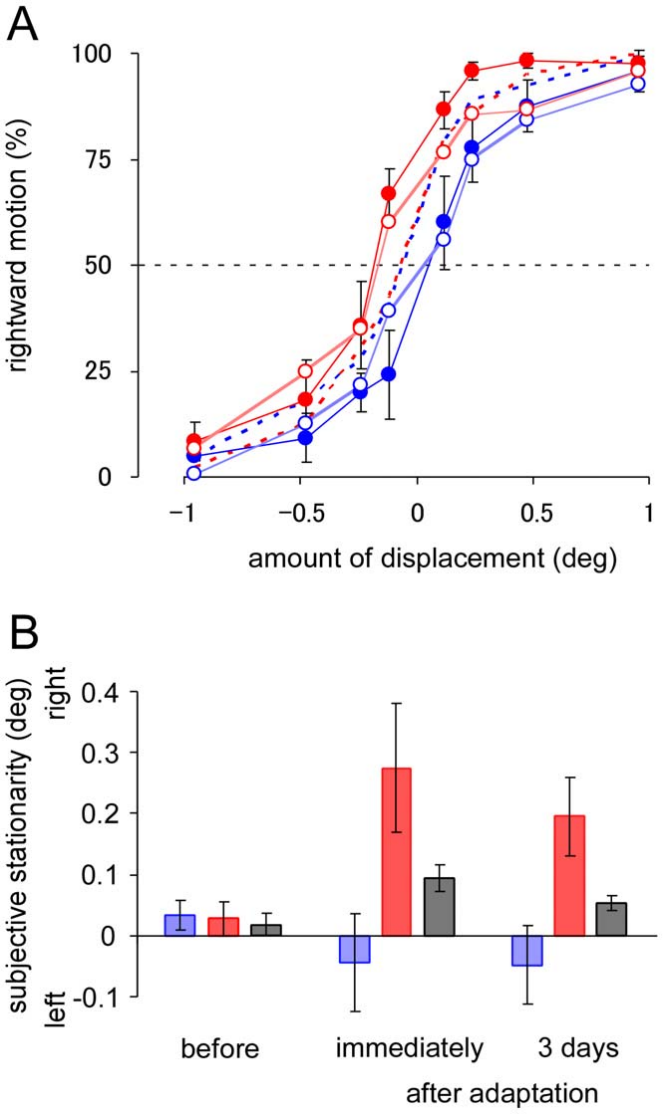
Sound-contingent visual motion aftereffects. (A) The proportion of rightward motion perception of visual stimuli as a function of the amount of physical displacements of visual stimuli. Positive values indicate rightward visual motion in the horizontal axis and negative values leftward visual motion. Blue lines represent the results for leftward sound condition and red lines rightward sound condition. Dashed lines represent the results obtained before adaptation. Filled circles represent the results obtained immediately after adaptation and open circles 3 days after adaptation. (B) The points of subjective stationarity (PSS). Positive values represent the shift of PSS in the direction of the leftward visual motion. Blue bars represent the results for leftward sound condition, red bars rightward sound condition, and gray bars no sound condition. Error bar denotes the standard error of the mean.

Before the exposure to apparent motion, sounds did not affect visual motion perception. However, sounds acquired driving effects for visual motion after the exposure. The PSS shifted in the direction of the leftward visual motion, when the first visual stimulus was synchronized with the tone that was accompanied with the leftward stimulus during the exposure and the second stimulus with the tone that was accompanied with the rightward stimulus (rightward sound condition). On the contrary, the PSS shifted in the direction of the rightward visual motion, when the sound sequence was reversed (leftward sound condition) (Fig. 2a). Of special interest was that the effect was clearly observed even 3 days after the exposure (Fig. 2b). A two-way analysis of variance (ANOVA) showed the PSSs are significantly different for sound conditions (*F*_2,20_ = 5.502, *p*<0.05) and the exposure is significantly effective (*F*_2,20_ = 5.217, *p*<0.05), and that the interaction between the exposure and the sound condition is significant (*F*_4,20_ = 3.788, *p*<0.05). Post hoc tests (Tukey's HSD) revealed the significant differences in PSS between the rightward and leftward sound conditions measured immediately after the exposure (*p*<0.05) as well as 3 days after the exposure (*p*<0.05). These results indicate that, after prolonged exposure to visual apparent motion with tones, the tones became drivers for illusory motion perception.

**Figure 3:**
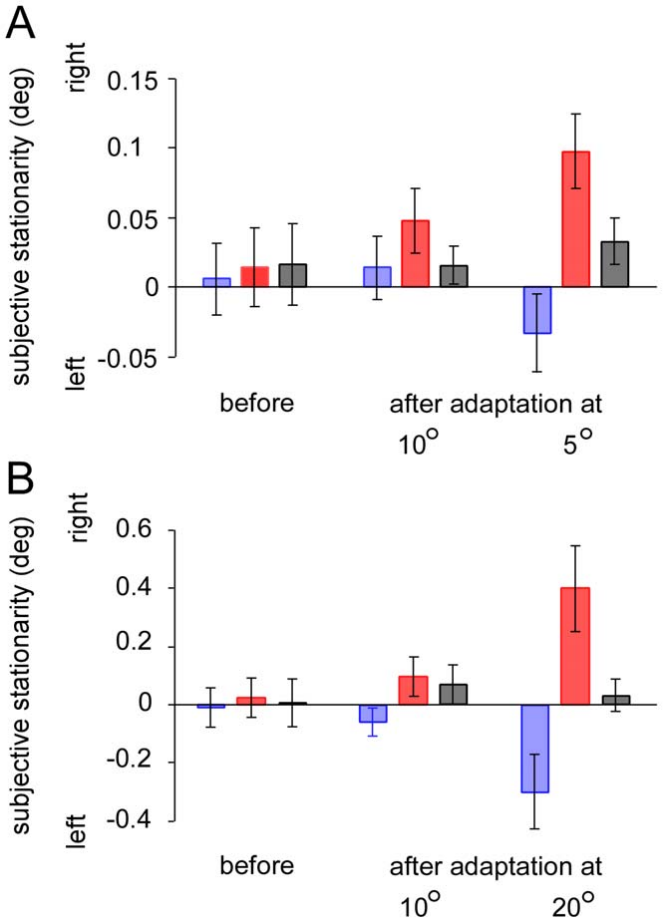
Points of subjective stationarity. (A) Results measured at 5° right of the fixation. (B) Results measured at 20° right of the fixation. Positive values represent the shift of PSS in the direction of the leftward visual motion. Blue bars represent the results for leftward sound condition, red bars rightward sound condition, and gray bars no sound condition. Error bar denotes the standard error of the mean.

Next, we tested whether the effect was observed at retinal positions that were not exposed to the apparent motion with tone bursts. Before and after the exposure to the apparent motion around 10.0° right from the fixation, the same tests were carried out at 5.0° and 20.0° right from the fixation. We thought that the audiovisual aftereffect might be mediated in higher cortical areas where cells receive both visual and auditory inputs and have large receptive fields providing the neural substrates for translational invariance. We therefore expected that the effect would be observed even in retinal positions other than the exposed positions. However, the effect was not observed at 5.0° and 20.0° right of fixation, when subjects were exposed to the apparent motion with tone bursts at 10.0° right from the fixation, implying a fairly rigid localization of the aftereffect. Indeed, notable aftereffects were observed at 5.0° and 20.0° right of fixation, when exposure to the apparent motion was given at 5.0° and 20.0° right of fixation respectively (Fig. 3). A two-way ANOVA showed that the PSSs are significantly different for sound conditions (*F*_2,20_ = 8.369, *p*<0.01) and that the interaction between the exposure and the sound condition is significant (*F*_4,20_ = 4.873, *p*<0.01) when tested at 5.0°. Post hoc tests (Tukey's HSD) showed the significant differences in PSS between the rightward and leftward sound conditions measured after the exposure to the apparent motion at 5.0° (*p*<0.05). A two-way ANOVA also showed that the PSSs are significantly different for sound conditions (*F*_2,20_ = 8.988, *p*<0.01) and that the interaction between the exposure and the sound condition is significant (*F*_4,20_ = 5.428, *p*<0.01) when tested at 20.0°. Post hoc tests (Tukey's HSD) revealed the significant differences in PSS between the rightward and leftward sound conditions measured after the exposure to the apparent motion at 20.0° (*p*<0.05). These results indicate that the audiovisual aftereffect is well observed at the retinal position that is previously exposed to apparent motion with tone bursts.

**Figure 4:**
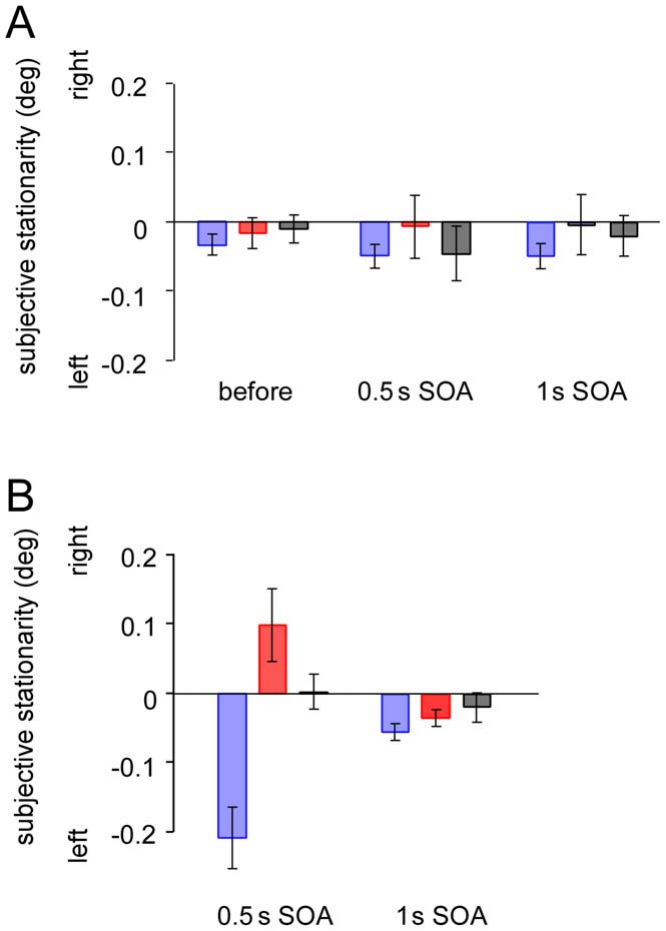
Points of subjective stationarity measured at 10° left of the fixation. (A) PSSs before and after adaptation to 1000 ms SOA. (B) PSSs after adaptation to 500 ms SOA. Positive values represent the shift of PSS in the direction of the leftward visual motion. Blue bars represent the results for leftward sound condition, red bars rightward sound condition, and gray bars no sound condition. Error bar denotes the standard error of the mean.

Auditory signals could modulate the perceived positions of visual inputs when the reliability of visual stimuli decreased [9]. It might be possible that sounds would be associated with the positional information of visual stimuli and, after forming the association, the sounds modulated the perceptual positions of the visual stimuli so that illusory visual apparent motion would be evoked. Indeed, it was shown that a blinking visual stimulus appears as lateral motion when the flash onset is synchronized to an alternating left-right sound source [5], [6]. We therefore tested whether motion perception was necessary for the aftereffect. As mentioned earlier, the aftereffect lasted for considerably long time but had rigid localization. We tested in the other visual hemifield. Exposure to the visual displacement with tones as well as test sessions was given at 10.0° left from the fixation. Subjects were exposed to the visual displacement with tones for 6 min. During this period, two white circles were presented in alternation at 7.5° and 12.5° left from the fixation, respectively. The duration of each circle was 400 ms and SOA was 1000 ms. The onsets of the two circles were synchronized to a tone burst of high and low frequency, respectively. In this condition, two circles were hardly perceived as a single circle moving side by side. Although the duration of the exposure was twice as long as that in the previous experiments, the PSS did not shift at all. We further tested with the same SOA of 1000 ms as the adaptation, but the PSS did not shift either, suggesting motion perception would be necessary for the aftereffect (Fig. 4a). Indeed, the PSS shifted considerably after exposure to the visual displacement with tone bursts where two white circles were presented with 500 ms of SOA so that visual apparent motion was consistently perceived (Fig. 4b). A two-way ANOVA showed that the PSSs are significantly different for sound conditions (*F*_2,20_ = 4.240, *p*<0.05) and that the interaction between the exposure and the sound condition is significant (*F*_4,20_ = 3.09, *p*<0.05). Post hoc tests (Tukey's HSD) showed the significant differences in PSS between the rightward and leftward sound conditions measured after the exposure to the apparent motion with 500 ms of SOA (*p*<0.05). However, the phenomena were not observed at all, when tested with the SOA of 1000 ms.

## Discussion

The present study clearly demonstrated that prolonged exposure to visual apparent motion with sounds results in a long-lasting aftereffect: a visual stimulus blinking at a fixed location was perceived as lateral motion in the same direction as the previously exposed apparent motion, when the flash onset was synchronized to the sounds.

It was demonstrated that auditory spatial cue induces the motion perception of a visual stimulus away from the cued location [2]. An auditory attentional cue might induce the illusory visual motion since the visual percept for the stimulus might gradually develop from the cued side. It might be also possible that the alternation of sound frequency would induce eye movements. However, if these ware the cases, the motion illusion should be observed at retinal positions that had not been exposed to apparent motion with tones. Indeed, there were no difference between eye movements measured before and after the adaptation and no relationship between eye movements and subjective judgments (Figure S1). Furthermore, the rigid localization of the aftereffect ruled out the possibility that the phenomena might result from response biases.

Although spatial superiority of vision over audition has been confirmed in motion processing [10]–[12], it was recently reported that the alternation of sound location could induce the illusory visual motion perception of static visual stimuli in peripheral visual field [5], [6]. The present study showed that sounds without explicit spatiotemporal information are able to trigger visual motion perception for a static visual object. This sound-induced illusory visual motion is observed only when a visual stimulus is presented in conjunction with sounds, and has very similar properties as those of contingent aftereffects.

Contingent motion aftereffects have been demonstrated in visual [13] and auditory domains [14]. It was shown that visual motion information strongly influences the contingent auditory motion aftereffect [15]. These ubiquitous phenomena are well known for their long lasting effects. The sound-induced illusory visual motion also persisted for an extremely long time. However, the sound-induced motion on appearance was positive, whereas most contingent aftereffects were negative in direction to the adaptation stimulus: after exposure to repeated alternations of a red contracting spiral and a green expanding spiral, the red stationary spiral appeared to be expanding and the green stationary spiral contracting [12]. The underlying mechanisms of the sound-induced motion would be quite different from those of contingent aftereffects.

It was recently shown that the visual system can be conditioned to use visual cues for the perception of a bistable stimulus [16], [17]. In these cue recruitment experiments, subjects were exposed to a rotating Necker cube, the perceived direction of which was forced by depth cues. An extraneous signal was also presented, contingent upon the direction of rotation. After 45 min exposure to this stimulus pattern, the extraneous signal acquired the same effect as the depth cues that were contingent during the exposure. It was also shown that the effectiveness of the signal was long lasting [16] and well observed at the exposed retinal position [17]. These properties are very similar to the sound-induced illusory visual motion. However, there is a remarkable difference in exposure time: a few minutes exposure was sufficient for the sound-induced motion, whereas 45 min exposure was necessary for the cue recruitment. Recent behavioral studies showed that novel multisensory associations can develop very rapidly [18], [19]. It is very likely that the strong association between sound sequence and visual motion is easily formed within a short period and that, after forming the association, sounds are able to induce illusory visual motion perception for a static visual object.

## Materials and Methods

The experiments were approved by the local ethics committee of Tohoku University. Written consent was obtained from each participant prior to the experiments.

Six observers including authors had normal or corrected-to-normal vision and normal hearing. The visual stimuli were presented on a 24 inch CRT display (refresh rate: 60 Hz) with viewing distance of 1 m. A red circle (0.4° in diameter; 17.47 cd/m^2^) for fixation and white circles (5.12 cd/m^2^, 1.0° in diameter) were presented on a black background. Tone bursts (85 dB SPL, 50 ms in duration with 5 ms rise and fall time) were generated digitally (sampling frequency 44.1 kHz), delivered by headphones. We confirmed that the onset of the visual and the auditory stimuli was synchronized using a digital oscilloscope. The observers were instructed to place their heads on a chin rest. All the experiments were conducted in a dark room.

PSSs were measured before and after subjects were exposed to visual apparent motion with tone bursts. In exposure phase, two white circles were placed side by side and presented in alternation. The distance between the two circles was 5.0°. The center between two circles was 5.0°,10.0°, or 20.0° right or left of fixation. The duration of each circle was 400 ms and stimulus onset asynchrony was 500 ms or 1000 ms. For half of the subjects, the onset of the leftward circle was synchronized to a tone burst of high (2 kHz) frequency and the rightward circle to a low (500 Hz) frequency tone. For the remaining half, the relationship was reversed. Subjects were asked to keep looking at the fixation and exposed to the visual apparent motion with tone bursts for 3 min.

In test sessions, two circles (with 400 ms duration in each) were presented with 500 ms or 1000 ms of SOA, synchronized with two tone bursts. In a rightward sound condition, the first visual stimulus was synchronized with the tone that was accompanied with the leftward stimulus during the exposure to apparent motion and the second stimulus with the tone that was accompanied with the rightward stimulus. In a leftward sound condition, the relationship was reversed. The no-sound condition was also included. The visual stimulus was displaced 0.06°, 0.12°, 0.24°, or 0.48° from left to right or vice versa when tested at 5.0°. 0.12°, 0.24°, 0.48°, or 0.96° at 10.0°, and 0.24°, 0.48°, 0.96°, or 1.92° at 20.0°. The amount of displacement and the sound condition were randomized from trial to trial. The observers were asked to judge whether the visual stimulus moved leftward or rightward. 20 responses were obtained for each condition.

## Supporting Information

Figure S1 Eye movement in the horizontal direction. The movement of right eye was recorded using infrared reflective oculography. Eye movements before (A) and after (B) the adaptation. Traces represent the movements for the last 10 trials where an observer judged that the visual stimulus moved rightward in the rightward sound condition. (C) Eye movements after the adaptation. Traces represent the movements for the last 10 trials where the observer judged that the visual stimulus moved leftward in the rightward sound condition. Vertical lines represent the onset of the visual stimulus.
(0.80 MB TIF)

Video S1 Test Stimulus. Demonstration of test stimuli. A circle is blinking at a fixed location and each onset is synchronized to tones of alternating frequencies.
(2.87 MB MOV)

Video S2 Adapting Stimulus. Demonstration of adapting stimulus. Two white circles placed side by side are presented in alternation, producing visual apparent motion perception. The onsets of the two circles are synchronized to a tone burst of high (2 kHz) and low (500 Hz) frequency, respectively.
(1.77 MB ZIP)
