# Supplementary figures and images for: Sounds Move a Static Visual Object
Source: PLoS One. 2010 Aug 19;5(8):e12255. doi: 10.1371/journal.pone.0012255 (PMC2924383; doi:10.1371/journal.pone.0012255)

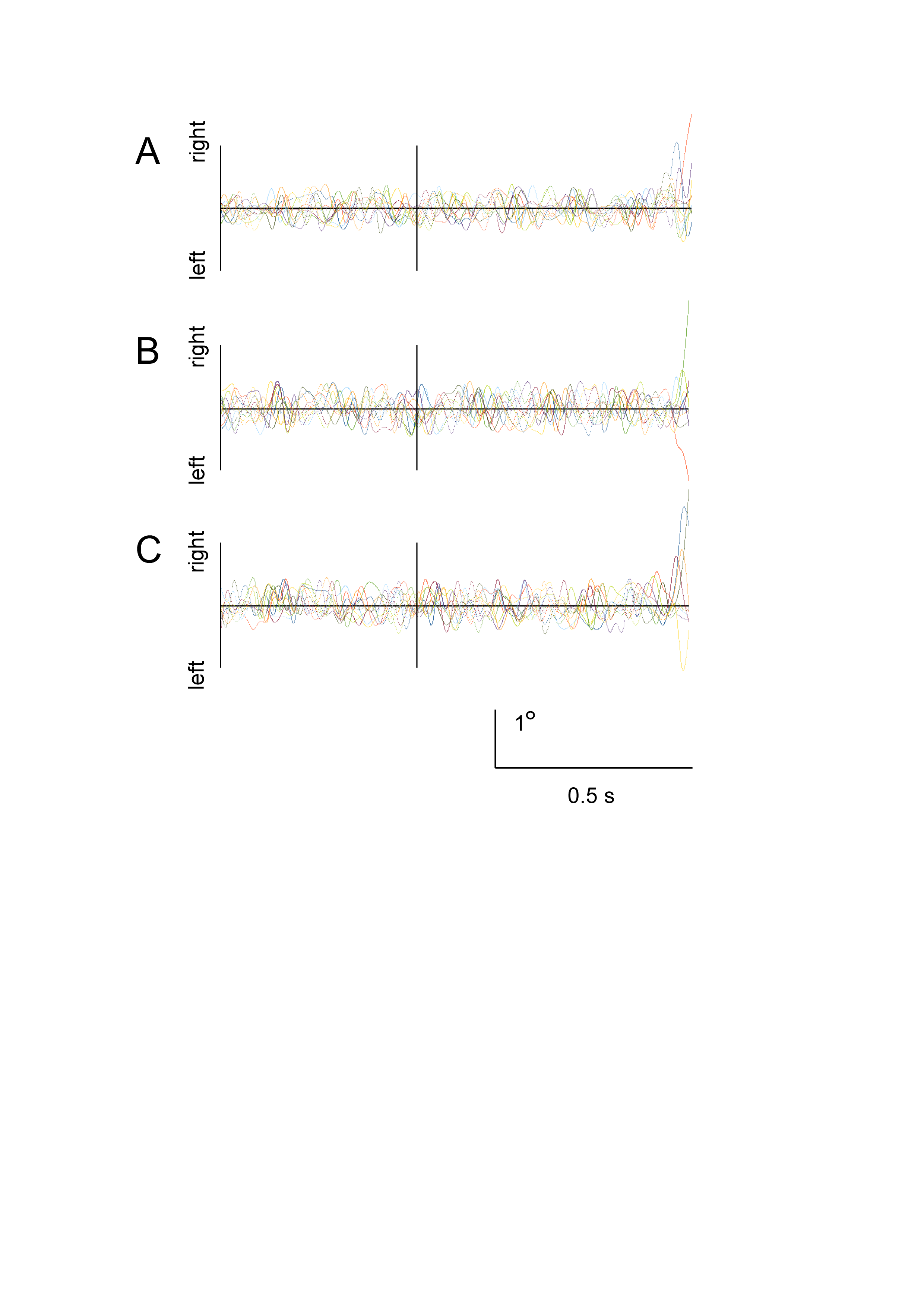

Supplement: Figure S1 — Eye movement in the horizontal direction. The movement of right eye was recorded using infrared reflective oculography. Eye movements before (A) and after (B) the adaptation. Traces represent the movements for the last 10 trials where an observer judged that the visual stimulus moved rightward in the rightward sound condition. (C) Eye movements after the adaptation. Traces represent the movements for the last 10 trials where the observer judged that the visual stimulus moved leftward in the rightward sound condition. Vertical lines represent the onset of the visual stimulus. (0.80 MB TIF) [file pone.0012255.s001.tif]
